# Oral medicine acceptance in infants and toddlers: measurement properties of the caregiver-administered Children’s acceptance tool (CareCAT)

**DOI:** 10.1186/s12887-018-1080-4

**Published:** 2018-03-22

**Authors:** Joern Blume, Ana Lorena Ruano, Siri Wang, Debra J. Jackson, Thorkild Tylleskär, Liv Inger Strand

**Affiliations:** 1 Centre for International Health, University of Bergen, Postbox 7804, N-5020 Bergen, Norway; 2 School of Public Health, University of the Western Cape, Cape Town, South Africa; 3 Center for the Study of Equity and Governance in Health Systems, Guatemala City, Guatemala; 4 Norwegian Medicines Agency, Oslo, Norway; 5 Department of Global Public Health and Primary Care, University of Bergen, Bergen, Norway

**Keywords:** Acceptance, Acceptability, Behavior, Child, Children under 5 years, Oral medicine, Medication, Reliability, Informant-report

## Abstract

**Background:**

Developing age-appropriate medications remains a challenge in particular for the population of infants and toddlers, as they are not able to reliably self-report if they would accept and consequently take an oral medicine. Therefore, it is common to use caregivers as proxies when assessing medicine acceptance. The outcome measures used in this research field differ and most importantly lack validation, implying a persisting gap in knowledge and controversy in the field. The newly developed Caregiver-administered Children’s Acceptance Tool (CareCAT) is based on a 5-point nominal scale, with descriptors of medication acceptance behavior. This cross-sectional study assessed the measurement properties of the tool with regards to the user’s understanding and its intra- and inter-rater reliability.

**Methods:**

Participating caregivers were enrolled at a primary healthcare facility where their children (median age 6 months) had been prescribed oral antibiotics. Caregivers, trained observers and the tool developer observed and scored on the CareCAT tool what behavior children exhibited when receiving the medicine (*n* = 104). The video-records of this process served as replicate observations (*n* = 69). After using the tool caregivers were asked to explain their observations and the tool descriptors in their own words. The tool’s reliability was assessed by percentage agreement and Cohen’s unweighted kappa coefficients of agreement for nominal scales.

**Results:**

The study found that caregivers using CareCAT had a satisfactory understanding of the tool’s descriptors. Using its dichotomized scores the tool reliably was strong for acceptance behavior (agreement inter-rater 84–88%, kappa 0.66–0.76; intra-rater 87–89%, kappa 0.68–0.72) and completeness of medicine ingestion (agreement inter-rater 82–86%, kappa 0.59–0.67; intra-rater 85–93%, kappa 0.50–0.70).

**Conclusions:**

The CareCAT is a low-cost, easy-to-use and reliable instrument, which is relevant to assess acceptance behavior and completeness of medicine ingestion, both of which are of significant importance for developing age-appropriate medications in infants and toddlers.

**Electronic supplementary material:**

The online version of this article (10.1186/s12887-018-1080-4) contains supplementary material, which is available to authorized users.

## Background

There is a move towards patient-centered development of formulations for pediatric oral medicines, reflected in legislation from both the European Medicines Agency (EMA) and the US Food and Drug Administration (FDA). As a consequence, pharmaceutical companies are now required to provide a clear strategy for the development of pediatric formulations for relevant new medication to be marketed, describing how to ensure its age-appropriateness [1, 2]. However, regulators have been criticized for not providing evidence-based guidance on the acceptability aspects [3]. This area is still evolving, and there is a need to provide evidence on perceptions of the relevant stakeholders, i.e. the children and their caregivers as the end-users of oral medicines.

The term *acceptance* is commonly defined as “the overall ability of the patient and caregiver to use a medicinal product as intended” [4]. However, a more operational definition is warranted as a basis for age-appropriate outcome measures in infants and toddlers. Until recently, research tended to be more generalized about a very heterogeneous group of children, and the youngest children have rarely been studied. In addition, over the past three decades, research focus has shifted between taste, palatability and swallowability, all of which are components of today’s understanding of acceptance [5–7]. Furthermore, in pediatric practice worldwide, prescriptions of oral medicines for the youngest children remain to be driven by the availability of formulations [8], rather than by considering any age-specific preference or needs of the children.

It is particularly challenging to determine whether an oral medicine is accepted by infants and toddlers, which might only be assessed indirectly by observation. Children under the age of 4 years cannot reliably self-report an outcome, such as acceptance. For this age group informant-reports are used, most commonly of caregivers as proxies [9, 10]. The different ways to report outcomes, e.g., time to administer a medicine [11], the completeness of its ingestion [12], or a child’s acceptance decided by the proxy [13–15], make comparison of assessments difficult. The lack of well-designed and age-appropriate instruments is recognized [16, 17]. There is a growing need to evaluate children’s acceptance of oral medicines, both during development of new ones and also for medicines already on the market. Success or failure of the treatment is ultimately impacted by behaviors of children taking the medicines. In this context, we have developed the Caregiver-administered Children’s Acceptance Tool (CareCAT). In accordance with current guidelines suggesting proxies to report observational content, CareCAT assesses the acceptance of oral medicines based on observed child behavior [9]. We have used this new instrument to separately assess acceptance behavior and completeness of medicine ingestion in infants and toddlers receiving oral liquid antibiotics. The study aimed to assess the measurement properties of the CareCAT tool with regards to the user’s understanding and its intra- and inter-rater reliability. The recommended guidelines of the EQUATOR network for reporting reliability and agreement studies (GRRAS) were adopted [18].

## Methods

### The tool

**Fig. 1:**
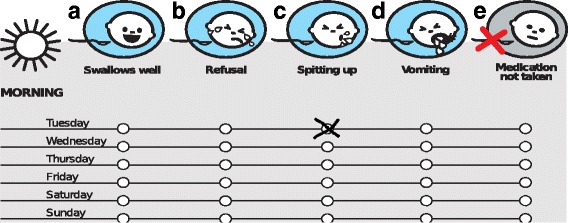
CareCAT report of a child spitting when receiving oral medication on a Tuesday morning

The CareCAT tool enables longitudinal measurements of behavioral responses during a treatment for up to 4 weeks. It is a single page diary with a 5-point nominal scale, which provides one descriptor of positive acceptance behavior; ‘swallows well’ and four descriptors of negative acceptance behavior, i.e. ‘refusal’, ‘spitting’, ‘vomiting’, and ‘medication not taken’. ‘Swallows well’ characterizes that the child received and retained the oral medicine. A child’s ‘refusal’ refers to behavior hampering the medicine reception partly or as for ‘medication not taken’ completely when a child is totally unwilling. The descriptors ‘spitting up’ and ‘vomiting’ are options for behaviors indicating that the medicine has been received but not completely been retained. Each of these descriptors is shown along with a pictogram representing it (Fig. 1). After each medicine administration the user reports observed behavior by ticking boxes that correspond to the point in time when the medicine was administered as well as to the relevant descriptor(s). As they are not mutually exclusive, descriptors (one or several) are chosen that best represent the child’s behavior. A completed diary may be interpreted by tallying the scores and generate proportions of how often one or several descriptors were reported out of all events of medicine administration. The scores can provide information on a child’s acceptance behavior and also indirectly inform about the number of potentially incomplete ingestions.

The CareCAT is intended to be utilized in clinical practice when a detailed picture of behavioral challenges during a child’s treatment period is warranted. In a clinical trial, it might allow to estimate the general acceptance of a medication in a population of children of different ages. The CareCAT has been designed to be administered by caregivers in their home environment but can also be used by health professionals. Informants require basic literacy and numeracy skills for the tool to be correctly used.

### Design

This cross-sectional study examines inter-rater and intra-rater reliability of the CareCAT tool when used by caregivers of children under the age of 5 years receiving oral antibiotics. It also explores the users’ understanding of the tool descriptors, an aspect of content validity.

#### Sample size

Following recommendations for reliability studies by the consensus-based standards for the selection of health measurement instruments (COSMIN) group, we chose a sample size of at least 50 observations [19]. However, it was deemed important that enrolment continued until all descriptors of the tool had been reported at least once. In particular, the tool descriptors ‘vomiting’ and ‘medication not taken’ were assumed to be rarely observed, based on clinical experience and the reports of Marshall et al. [20].

#### Setting

Participants were recruited at a primary healthcare facility in Mitchells Plain, Cape Town, South Africa, where children received one of eight antibiotics as treatment of a current sickness or as a prophylaxis. Procedures took place in a neutral undisturbed area inside the health facility.

#### Sampling

We collected reliability data from caregivers who administered the medicine to their child as well as from the research assistants, and the tool developer who observed the process. Caregivers were recruited through purposive consecutive sampling. When a nurse at the health facility had routinely prescribed an oral antibiotic to a child, she identified the caregiver as a potential participant. Caregivers were eligible if: a) they were above the legal age of 18 years and the legal guardian of the child; b) the child was less than 5 years of age and had been prescribed oral liquid antibiotic treatment; c) they were willing to administer the first dose of treatment in the presence of an observer. Caregivers were not eligible if: a) they were not sufficiently familiar with Xhosa or English, the languages predominantly spoken in the area, for which the study materials were available; b) they had participated in the present study before; and c) the child needed hospitalization as judged by a clinic nurse. Three research assistants, here called “observers”, aged 19, 30 and 40 years were trained in how to score with the tool, as well as how to introduce it to the participants and how to interview them. All were confident and fluent in both Xhosa and English, and had completed secondary school education. They as well as, JB, a pediatrician and the first author who developed the tool, here called “tool developer”, served as external observers.

### Data collection

Data were collected between April and June 2016. As the first step, we obtained informed consent, after which we gathered information about the child’s medication and age, as well as the caregiver’s age, language, education and socio-economic status. The participant was then introduced to the tool through a standardized protocol aimed at minimizing bias. Detailed explanations were given about the five descriptors and the time-structure of the tool, after which the reporting process had to be practiced using six given real-life examples (Additional file 1). This procedure was repeated until all these examples had been accurately scored. Subsequently, the caregiver administered one dose of the oral antibiotic to the child, which was video-recorded using a smart phone. The caregiver and the external observer(s) independently scored their observations on the CareCAT tool. To minimize bias, caregivers were not made aware that their scores would be compared with those of the observers. The scoring was done individually without any communication between the assessors. After having scored on the tool, we asked caregivers to describe in their own words what they had seen their child doing, and how they would explain each descriptor. The three observers were also requested to score a sequence of video clips of children receiving oral medicine that had been recorded during the study. The videos were shown in two rounds, first in a systematic consecutive order, and second in a randomized order. To minimize recall bias at least five days had to pass between the day of administration and the first video-view, and at least 3 days between the first and second video-view.

General participant information, CareCAT scores of the different users, as well as the caregivers’ explanation of the tool descriptors were captured and quality checked using EpiData Entry software 3.1. Interviews were tape-recorded, consecutively transcribed in Xhosa or English, and the Xhosa transcriptions subsequently translated into English. After reading through the complete transcripts, we captured a summary of the individual explanations of the tool descriptors.

### Data analysis

The measurement properties of the CareCAT tool were examined by: 1) exploring similarities and differences in scoring patterns among the different users, 2) assessing the agreement of scores between different users (inter-rater) and the reproducibility of scores by the observers (intra-rater), and 3) evaluating the caregivers’ understanding of each tool descriptor by reviewing their individual explanations. Scoring patterns of caregivers, observers and tool developer were reported descriptively. The user’s scores were analyzed in 2 groups, first as raw scores called ‘detailed scores’, and second after having divided them into 2 categories called ‘dichotomized scores’. The dichotomization was done firstly with regards to the child’s ‘acceptance behavior’ (positive/negative), which was considered positive if a child’s behavior was scored solely ‘swallows well’; and negative for all other scoring categories. We secondly categorized the scores focusing on whether the oral medicine had been received and retained by the child entirely or not, here called ‘medicine ingestion’ (complete/incomplete). We assumed that scoring ‘refusal’ and ‘swallows well’ in combination represented a child showing dislike, but still swallowing the medicine while the combination of ‘swallows well’ with ‘spitting up’ and/or ‘vomiting’ indicated some loss of medicine. Therefore, a medicine ingestion was considered complete if scored ‘swallows well’ alone or in combination with ‘refusal’, and incomplete for all other scoring categories. To assess the tool’s reliability, we cross-tabulated scores and calculated percentage agreement and unweighted Cohen’s kappa (κ) coefficients of agreement (with 95% confidence intervals, 95% CI) for nominal scales, using statistical software package of SPSS 23 and Microsoft Excel. While percent agreement represents the proportion of scores classified into the same categories by either two users or replicate observations of the same user, Kappa statistic measures the frequency of exact agreement while discounting ‘the proportion of agreement expected by chance alone’ [21, 22]. We further categorized Kappa values according to Landis and Koch’s criteria: as ‘poor’ if less than 0.2, ‘fair’ if between 0.21 and 0.4, ‘moderate’ if between 0.41 and 0.61, and ‘strong’ if above 0.61 [23]. Cross-tabulations were used to compare the agreement of scores between the users of the tool. The caregivers’ scores refer to multiple caregivers of which each solely scored their own child. The observer’s score refers to scores of three different observers, of whom only the score of the observer who introduced and instructed the caregiver was used. The summarized explanations of each tool descriptor given by caregivers were first coded; later codes were merged to main categories of themes that were displayed in a table, with examples of participants’ quotes.

### Ethical considerations

The study was approved by the University of Western Cape’s Faculty Research Committee and the City of Cape Town, South Africa. Participants gave written consent for their participation. A separate consent form was used for the video-recording of the participant’s child receiving the medicine. Participants were informed that the material would be reviewed and the children’s behavior scored within the study team to determine whether CareCAT is used always in the same way.

## Results

**Fig. 2:**
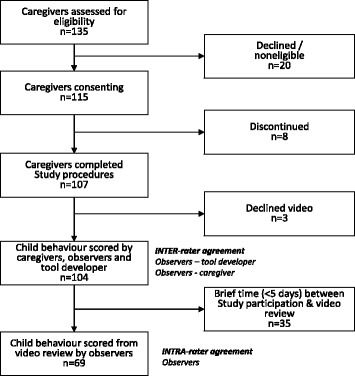
Study profile to determine measurement properties of CareCAT

We enrolled 115 caregivers, of whom 104 completed the study, and whose children’s behavior during the medicine administration was scored by the caregiver, an observer and the tool developer (Fig. 2). Caregivers were mostly mothers of the children (94%), with median age of 29 years (interquartile range, IQR 25;33), most (95%) having had secondary school experience. The children’s median age was 6 months (IQR 2;15), 57% being girls (Table 1). As part of the reproducibility assessment, the observers scored 69 video-recordings of children receiving oral medicine.

**Table 1:** Baseline characteristics of participating caregivers and observed children

Caregivers	n = 104
Relation to child	
Mother	99
Father	2
Grandmother/aunt	3
Mother tongue	
Xhosa	97
Zulu	2
Afrikaans	1
Shona / Chichewa	4
Highest level of education	
Some primary	5
Some secondary	55
Up to grade 12	12
Matric (A-levels)	32
Living conditions	
Electricity at dwelling	101
Drinking water nearby	54
Dwelling’s walls made of bricks	26
Children	n = 104
Age	
1–3 months	38
4–6 months	18
7–9 months	9
10–11 months	8
12–23 months	14
2–4 years	17
Oral antibiotics received	
Amoxicillin	51
Trimethoprim-Sulfamethoxaxole	36
Cephalexin	7
Erythromycin	3
Ciprofloxacin	2
Metronidazol	2
Phenoxymethyl Penicillin	2
Amoxicillin-Clavulanic Acid	1

### Scoring patterns among the different users

The scoring of caregivers, observers and the tool developer resulted in a total of 12 scoring categories (Table 2), with the scoring pattern of the caregivers being slightly different from that of the observers and the tool developer. While the caregivers predominantly chose to report their observation in the form of a single score (88%), this was less frequent among the observers and the tool developer (58% and 62%, respectively). For example, caregivers reported ‘refusal’ alone in 11% of the administrations; but ‘refusal’ and ‘swallows well’ for 5%. In contrast, the observers reported ‘refusal’ alone in 1% and ‘refusal’ in combination with ‘swallows well’ in 16%. ‘Vomiting’ and ‘medication not taken’ were rare and usually reported in combination with ‘spitting’ and/or ‘refusal’ (Table 2).

**Table 2:** Patterns of scoring categories according to CareCAT user

			Caregivers N = 104 %	Observers N = 104 %	Tool developer N = 104 %
1. Single score	Swallows well		71	57	59
2. Single score		Refusal	11	1	2
3. Single score		Spitting	5		1
4. Single score		Medication not taken	1		
5. Multiple scores	Swallows well	Refusal	5	16	9
6. Multiple scores	Swallows well	Spitting	3	18	20
7. Multiple scores	Swallows well	Refusal Spitting		4	2
8. Multiple scores		Refusal Spitting	3	3	3
9. Multiple scores		Refusal Vomiting	1		
10. Multiple scores		Refusal Spitting Vomiting		1	1
11. Multiple scores		Refusal Spitting Medication not taken			2
12. Multiple scores		Spitting Medication not taken			1

### CareCAT’s reliability based on the dichotomized scores

After dichotomizing the scores into positive or negative acceptance behavior, the CareCAT tool’s intra- and inter-rater agreement proved strong, irrespective of who was the user (Table 3). Importantly, 15% of the caregivers’ scores categorized as positive acceptance behavior were categorized as negative by the observers. The opposite – acceptance behavior categorized as positive by the observer’s but negative by the caregiver’s score – was rare (1%).

**Table 3:** *Reliability* of the CareCAT tool

	Intra-rater agreement (video-review^1^)			Inter-rater agreement^2^	
	Observer I	Observer II	Observer III	Tool developer	Caregivers
	N = 69	N = 69	N = 69	N = 104	*N* = 104
Scores dichotomized regarding acceptance behaviour^**3**^					
%^a^	89%	87%	87%	88%	84%
к^b^	0.68	0.72	0.69	0.76	0.66
95% CI^c^	0.47–0.88	0.54–0.89	0.51–0.87	0.64–0.89	0.52–0.80
interpretation	strong	strong	strong	strong	strong
Scores dichotomized regarding completeness of medicine ingestion^**4**^					
%^a^	93%	85%	91%	86%	82%
к^b^	0.67	0.50	0.70	0.67	0.59
95% CI^c^	0.39–0.94	0.23–0.76	0.48–0.92	0.52–0.83	0.42–0.76
interpretation	strong	moderate	strong	strong	moderate
*Detailed scores*					
%^a^	81%	73%	78%	75%	63%
к^b^	0.56	0.49	0.55	0.59	0.34
95% CI^c^	0.36–0.75	0.33–0.65	0.38–0.72	0.47–0.71	0.25–0.44
interpretation	moderate	moderate	moderate	moderate	fair

^a^agreement in %

^b^Cohen’s kappa coefficient

^c^95% confidence interval of kappa

^1^Comparison of scoring videos shown in systematic consecutive vs. random order

^2^Observers’ scores compared with scores of tool developer and caregivers

^3^Acceptance behavior: *positive*: swallows well vs. *negative*: all other combinations

^4^Medicine ingestion: *complete*: swallows well, also combined with refusal vs. *incomplete*: all other combinations

When the scores were dichotomized on the basis of complete or incomplete medicine ingestion, the inter-rater agreement between the caregivers and observers was moderate (Table 3). There were a few occasions in which the ingestion of medicine was categorized as incomplete by the caregivers’ scores, but as complete by observers, and vice-versa (8 and 10%). Inter-rater agreement between observers and tool developer was strong, whereas the intra-rater agreement of the observers varied slightly between moderate and strong.

### CareCAT’s reliability based on detailed scores

#### Caregiver versus observer

**Fig. 3:**
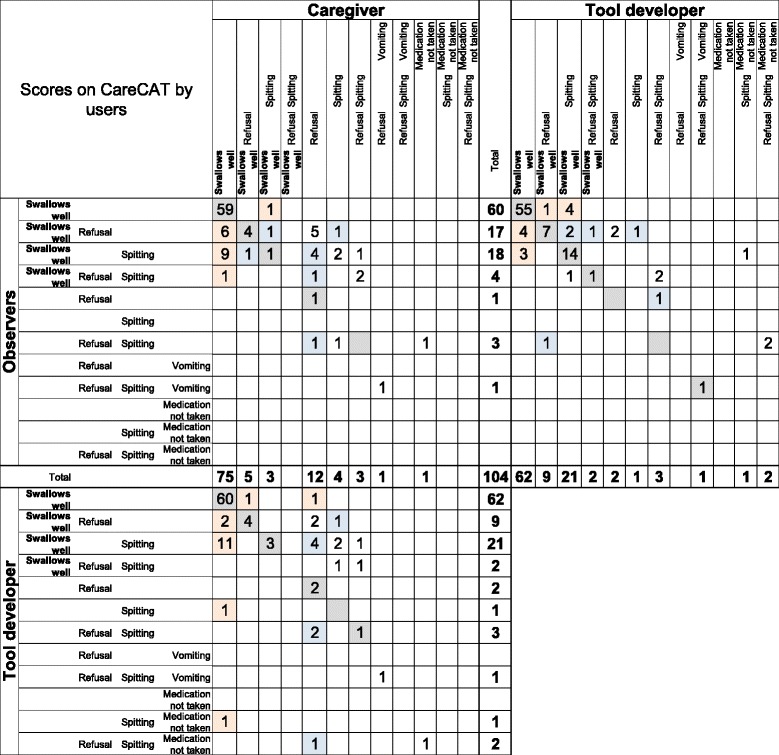
Cross-tabulation of detailed CareCAT scores by different users. Colored: scoring categories used by both users (grey); patterns of discordance: one user scoring negative behavior whereas the other scored ‘swallows well’ (pink:); one user scoring ‘refusal’ whereas the other scored ‘spitting’ (blue)

There was substantial concordance between the observers’ detailed scores and those of the caregiver (63%), particularly when a child was scored as solely ‘swallows well’ (78–79%). Inter-rater agreement was fair (Table 3). In at least 20% of all the children in whom caregivers scored ‘swallows well’, the observers scored ‘spitting’ or ‘refusal’. The opposite – observers scoring ‘swallows well’ whereas the caregiver had ticked a negative behavior – occurred in very few cases (2–3%). In instances where both, the caregiver and the observer had reported a negative behavior, it was common that the caregiver scored ‘refusal’ whereas the observer scored ‘spitting’ (21%) for the same child (Fig. 3).

#### Observers’ reproducibility and agreement with the tool developer

The observers’ detailed scores were reproduced in 73 to 81% of the repeated video-views and concurred in 75% with the scores of the tool developer (Table 3). Intra-rater agreement of the observers’ and inter-rater agreement between the observers and the tool developer were moderate. Importantly, observers and tool developer mostly agreed (77–78%) on scoring a child to have swallowed well. In 12% of the children that were scored to have shown negative behavior (*n* = 42), the observer scored ‘refusal’ whereas the tool developer scored ‘spitting’ (Fig. 3).

### Caregivers’ understanding of the tool descriptors (aspect of content validity)

Examples of the caregivers’ explanations of the tool descriptors are presented in Table 4. The descriptor ‘swallows well’ was explained as the actual act of swallowing, as exemplified by one caregiver stating that her child would drink and swallow the medicine well similar to how the child usually eats. On the other hand, many caregivers referred to ‘swallows well’ as the absence of negative child behavior. A caregiver reported, for example, that the child would ‘not give me a hard time when she drinks the medicine’. The explanation of ‘refusal’ entailed physical action such as aiming to prevent - but also to reverse - the intake of medicine by spitting and induced vomiting. One caregiver reported that her child ‘…would beat the spoon with the medicine, close her mouth, move her body and cry’. While most caregivers generally defined ‘spitting’ or ‘vomiting’ correctly, few also mentioned the loss of medicine through ‘spilling’ or ‘overflow’. ‘Medication not taken’ was described as actions resulting in no intake of medicine.

**Table 4:** Examples of caregivers’ verbal explanations of the five CareCAT descriptors

Swallows well
- Observing the act of swallowing the medicine *‘[I see that she] drinks the medicine and swallows’*
- Ingesting the medicine in absence of negative behaviors *‘He swallows and does not give me a hard time’*
Refusal
- Defensive behavior preventing the intake of medicine *‘By pushing the spoon’ or ‘fights with her hands’ or ‘turns away her head’*
- Defensive behavior reversing the intake of medicine *‘She cries, moves her body and then vomits’*
Spitting
- Forcing the medicine out actively *‘He spits or maybe blows the medicine out’*
- Medicine passively leaving the mouth (‘overflow’ or ‘spilling’) *‘When the medicines runs down the mouth’*
Vomiting
- *‘She takes out the medicine after feeling nauseous, then vomits it with food’*
Medication not taken
- No oral intake of medicine *‘She does not want [to take the medicine] until the medicine did not get in [the mouth]’*
- Intake without ingesting *‘I have tried to give her but [the medicine] was still not swallowed’*

## Discussion

We have explored the measurement properties of the CareCAT tool, a newly developed informant-reported outcome instrument used for scoring behaviors that infants and young children display while receiving oral antibiotics. Our results show that the tool is a relevant and reliable instrument to assess acceptance behavior and completeness of medicine ingestion when using its dichotomized scores, irrespective of who is scoring the observation. Caregivers were able to understand and use the descriptors of the scale when scoring their child’s behavior.

To our knowledge, this is the first low-cost, easy-to-use informant-reported outcome instrument to assess medicine acceptance that has been tested for reliability and validity in infants and toddlers. If implemented in practice, it could be used to follow-up children on long-term medication as part of the evaluation of adherence to treatment. Clinicians could then further probe to specify the types and intensity of certain behaviors. In a clinical trial setting, CareCAT’s dichotomized scores might enable a systematic assessment of medicine acceptance and intake in a population of children; the detailed scores given herein may serve descriptive purposes. The tool may bring light into the behavioral component of children’s acceptance at the level of end-users. This can potentially be useful in establishing the link between acceptance and adherence, yet to be proven [24].

CareCAT’s reduced reliability based on detailed scores may partly be explained by the presence of individual thresholds in reporting negative child behavior, and by methodological challenges to assess reliability of a tool measuring children’s acceptance through a proxy. The kappa coefficient of the intra-rater agreement, ranging here from k = 0.49 to 0.72, is similar to that derived from a tool used in the CALF-study [25], where reliability was examined from the perspective of the observers. However, lacking a gold-standard, we went on to evaluate the tool’s reliability verifying inter-rater agreement, but were unable to compare our results with other studies due to lack of reporting. Notably, while a similar study on infant’s dietary acceptance found no differences in correlations of ratings on children done by a research assistant, their own caregivers or another caregiver [26], we found greater disparity of agreement between caregivers versus observers, compared to agreement between observers and the tool developer. One explanation for the discordance in scoring patters varying among users might be that detailed scoring of behavior varied depending on whether the tool user was familiar with the child or not. Unlike a person scoring an unknown child, caregivers might consider prior experience of certain behaviors in varying intensity. Consequently, they tended to have higher thresholds in reporting a child’s negative behavior than the observers. Here, it is important to note that an observer confirmed most of the instances where a caregiver had reported negative behavior. Another reason for the reduced reliability may lay in a known weakness of Cohen’s kappa, which gives credit only to full agreement and is sensitive to a higher number of scoring categories [22, 27]. As a consequence, we dichotomized the scores, after which a sufficient level of reliability of the tool, supported by high kappa coefficient, could be demonstrated.

By using a scale on which a user scores observable behaviors, CareCAT’s design aligns with the guidelines for the research on pediatric patient-reported outcome instruments [9]. We intentionally did not ask informants to evaluate the medicine or make inferences about the child’s subjective experience, such as stating pleasantness of the medicine on behalf of the child [13–15, 25, 28–30], which is a common approach that has been debated for decades in this field [28, 31], and has been discouraged by pharmaceutical regulatory authorities [32]. Taste preferences are subjective, and the often-used hedonic scale was validated to determine one’s own taste preference and not that of somebody else [33], which underlines the importance of determining inter-rater agreement. The evaluation of the caregivers’ understanding of the tool descriptors confirmed an overlap between the two descriptors ‘spitting’ and ‘refusal’, found also when cross-tabulating the detailed scores. We believe this can be addressed by stressing to users that ‘refusal’ and ‘spitting’ can be scored in parallel.

### Social desirability

Expecting acceptance data to be biased, e.g. by informants reporting in a socially desirable manner, we chose tool descriptors that would avert the focus of caregivers from feeling assessed in their ability to administer a medicine to paying attention to the actual behavior of the child. The tendency to put themselves, the child or the medicine into a favorable light might have affected caregivers’ and observers’ scores differently [34, 35]. It might have lead caregivers to report negative behavior with a higher threshold. A different scoring pattern of the observers with their tendency to report negative behavior in parallel with ‘swallows well’ might show their focus on the medicine intake, no matter if the child displayed negative behavior or not. Rephrasing the descriptor as ‘swallows well’ with ‘neither refusal, spitting nor vomiting observed’ is one possibility in controlling this element of reporting bias. Furthermore, the tool could provide two scales – one to report completeness of ingestion and another for child acceptance behavior. However, adjusting the tool might be at the expense of the tool’s simplicity, which consequently might require higher literacy and numeracy levels of the user population and more instructions to the user. We consider it a strength that the tool with its current design is not restricted to being used by health professionals only; indeed, the results show that caregivers with different educational level enrolled here could self-administer the tool.

Our approach to dichotomize the CareCAT scores separately for acceptance behavior or completeness of medicine ingestion, intentionally deviates from others [20]. We believe that focusing only on the completeness of medicine ingestion, irrespective of child’s behavior, might also show a tendency to report the acceptance of a medication in a desirable manner.

### Methodological considerations

This study has several strengths: first, by focusing only on the age group of infants and toddlers, detection of observations typical for this group increased [36]. Second, by testing validity and reliability, we were addressing the lack of non-validated tools in this field. Furthermore, the tool was tested in the key population, for which it was developed in a real-life setting. By enrolling caregivers who were casual attendees at a healthcare facility, we have demonstrated the tool’s use in assessing asymptomatic as well as sick children when receiving antibiotics prescribed in practice. With a sample size of more than 100 rated observations, it also fulfilled by far the minimum requirement of participants in reliability studies [19].

While this study focused on a relevant knowledge gap in the field of the use of medicines in pediatrics, it has some limitations. It took place at a healthcare facility and not in the natural home environment, which we believe could have been perceived as rather intrusive. Future research should involve the tool’s implementation at a caregiver’s home with completion on multiple occasions. Another limitation is that the diversity achieved in response was not as homogeneously distributed as desired. This is related to the frequency of behaviors, such as vomiting, which occurred rarely, as reported by others [20, 37], and particularly depends on the palatability of the medicine.

## Conclusions

The results show that CareCAT is a low-cost, easy-to-use and relevant informant-reported outcome instrument to assess the acceptance of oral medicines in infants and toddlers who are unable to verbally give their opinion about a medicine. Dichotomizing reported CareCAT scores on child behavior enables reliable measures of both acceptance behavior and completeness of medicine ingestion. Both are of significant importance for our main goal - to make the child receive and retain an oral medicine with sufficient ease.

## Additional file

Additional file 1: CareCAT – tool introduction standard. Standardized instructions used to introduce the tool to the study participants. (DOCX 38 kb)

## Abbreviations

CareCAT: Caregiver-administered Children’s Acceptance Tool
CI: Confidence interval
EMA: European Medicines Agency
FDA: US Food and Drug Administration
GRRAS: Guideline for reporting reliability and agreement studies
IQR: Interquartile range
