# Supplementary material for: Oral medicine acceptance in infants and toddlers: measurement properties of the caregiver-administered Children’s acceptance tool (CareCAT)
Source: BMC Pediatr. 2018 Mar 22;18:117. doi: 10.1186/s12887-018-1080-4 (PMC5863835; doi:10.1186/s12887-018-1080-4)
Supplement: Supplementary file 1 — CareCAT – tool introduction standard. Standardized instructions used to introduce the tool to the study participants. (DOCX 38 kb) [file 12887_2018_1080_MOESM1_ESM.docx]

# Supplementary file

## CareCAT– tool introduction standard

I. Explaining CareCAT’s diary structure

The introducing person points out:

1. Timing of medicine: explaining e.g. morning/ evening dose

2. Days and dates: showing where days/dates are written

3. Child-behaviours: naming the 5 given child-behaviours

II. APPLYING 3 examples of child- behaviours on CareCAT

The introducing person shows the caregiver stepwise (according to 1.-3. above) how to find and tick the corresponding box and marks it using the following examples:

Imagine it is

- Wednesday evening, the child is spitting up when you administer the medicine (The introducing person states: You look for the moon symbolising the evening dose, the day which is Wednesday and the behaviour which is ‘spitting up’)
- Thursday morning, the child swallowed well when you administer the medicine (The introducing person states: You look for the sun symbolising the morning dose, the day: which is Thursday and the behaviour which is ‘swallows well’)
- Friday evening, the child pushes the spoon with medicine away when you administer the medicine, force is used to get it in (The introducing person states: You look for the moon: symbolising the evening, the day which is Friday and the behaviour which is ‘refusal’)

III. DEFINING the CareCAT descriptors more detailed

The introducing person reads out explanations of the behavioural descriptors:

| \| **Child-behaviour** \| **You see:** \| \| --- \| --- \| \| Swallows well: \| Medicine is swallowed down \| \| Refusal: \| Resistance of the child (e.g. fighting, crying) \| \| Spitting up: \| Some medicine is coming out of the mouth immediately \| \| Vomiting: \| Within 30 minutes after swallowing the medicine the child brings up something [vomits] \| \| Medication not taken: \| No medicine went into the mouth of the child \| |
| --- | --- | --- | --- | --- | --- | --- | --- | --- | --- | --- | --- | --- |

IV. READING OUT the rules to fill CareCAT

The introducing person reads out:

You must fill this diary each time immediately after you have given the medication to your child. Tick the behaviour what describes best what you observed your child doing. If you have doubts you can tick 2 child-behaviours (or more).

V. USER PRACTICE

The introducing person provides 6 standard examples to the user who has to score them using CareCAT : e.g. Wednesday evening medication- my child spits up medicine etc.

In case any of the given examples was incorrectly filled the introducing person has to re-do introduction (point I to V).
